# A Novel Geometry-Based Approach to Infer Protein Interface Similarity

**DOI:** 10.1038/s41598-018-26497-z

**Published:** 2018-05-29

**Authors:** Inbal Budowski-Tal, Rachel Kolodny, Yael Mandel-Gutfreund

**Affiliations:** 1 Faculty of Biology, Technion, Israel Institute of Technology, Haifa, 3200003 Israel; 2 Department of Computer Science, University of Haifa, Mount Carmel, Haifa, 3498838 Israel

## Abstract

The protein interface is key to understand protein function, providing a vital insight on how proteins interact with each other and with other molecules. Over the years, many computational methods to compare protein structures were developed, yet evaluating interface similarity remains a very difficult task. Here, we present PatchBag – a geometry based method for efficient comparison of protein surfaces and interfaces. PatchBag is a Bag-Of-Words approach, which represents complex objects as vectors, enabling to search interface similarity in a highly efficient manner. Using a novel framework for evaluating interface similarity, we show that PatchBag performance is comparable to state-of-the-art alignment-based structural comparison methods. The great advantage of PatchBag is that it does not rely on sequence or fold information, thus enabling to detect similarities between interfaces in unrelated proteins. We propose that PatchBag can contribute to reveal novel evolutionary and functional relationships between protein interfaces.

## Introduction

Understanding protein function is one of the key challenges facing molecular biologists. Generally, the protein sequence of amino acids determines a fold in space according to the physiochemical attributes of its residues. The specific fold exposes some residues to the surface of the structure, while other residues remain buried. The exposed residues are the protein surface, which is the part of the protein that potentially interacts with other molecules, such as DNA, RNA, small ligands, or other proteins. The interacting molecule binds a specific part of the surface, the interface, which matches the molecule’s binding preferences in terms of structure and physiochemical properties, e.g. electrostatics and hydrophobicity. Therefore, the protein sequence, the fold, the surface, and the interface – are all tied to protein function.

The three-dimensional (3D) structure of proteins is highly conserved in evolution and provides important information about their function^1^. Hence, a classic approach to infer functional similarity is to use a structural alignment, which is the process of identifying two equally sized substructures that are geometrically similar. The output of a structural alignment is the aligned substructures, superimposed on one another. One can measure the root mean square deviation (RMSD) between the sub-structures, to quantify their divergence from one another. Among the widely used structural alignment tools are CE^2^, DALI^3^, and FATCAT^4^. Indeed, many methods for protein function prediction rely on finding structural neighbors^5–8^. However, it has been shown that in some cases different folds can share similar functions, and in other cases proteins of the same fold can have different functions, e.g., interact with different substrates^9^. In such cases, the protein surface can shed new light on the protein function. In particular, the protein interface that mediates the interactions with other molecules is of interest^10–13^. Indeed, it has been shown that protein interfaces are far more conserved than the protein surfaces^14^.

Several algorithms were developed to identify surface similarities, independent of the overall protein folds. These algorithms represent the surface shapes in various ways, such as Alpha Shapes and Delaunay Triangulations^15–17^, Three-Dimensional Zernike Descriptors (3DZD)^18–21^, or an unordered collection of the three-dimensional (3D) coordinates of the surface atoms^22,23^. Some of these algorithms were implemented for a fast 3D comparison of protein surfaces^24,25^. In addition, algorithms were developed dedicatedly to compare interfaces - the functional part of the surface. The available interface-comparison and interface clustering algorithms are usually limited to specific interface types, such as protein-binding interfaces^26–33^, ligand-binding interfaces^13,34–40^ or nucleic-acid-binding interfaces^41^. Other methods based on local structural surface comparison have been employed for pocket comparison^42^ and interface prediction, for instance, predicting protein-protein interacting residues^43^ or finding ligand binding sites^21,44^.

Here, we present a novel geometry-based approach, named PatchBag, for characterizing and comparing protein surfaces or sub-surfaces (interface) in an accurate and highly efficient manner. In contrast to other interface-comparison tools, Patchbag is applicable for comparing interfaces of any type, as it does not require any information from the specific partner of the protein of interest. PatchBag represents protein surfaces or interfaces as vectors that count the number of appearances of various geometrical types of small surface units, defined as surface patches. This is known as the bag-of-words approach (BOW), where the surface is described as an unordered collection of local features. The advantage of using BOWs is that it is magnitudes faster to compare vectors than to compare the original objects. Indeed, the BOW approach is extensively used in large search systems such as web search engines^45^. BOW algorithms were also used in the context of protein study, as for example in SVM-Fold^46^ to detect protein sequence homology, in FragBag^47^ to rapidly retrieve protein structures from the Protein Data Bank (PDB), and to discriminate native structures from structure prediction decoys^23^. In PatchBag, we define local surface patches by the coordinates of the C-alpha atom of an exposed residue and their nearest C-alpha atoms that are not consecutive along the protein chain.

We use PatchBag to model the interfaces of 1,070 protein domains from the database of three-dimensional interacting domains (3DID)^48^. To test PatchBag’s ability to identify homologous interfaces we construct a homology-based network, connecting protein domains based on their PatchBag similarity of their known interfaces. We show that the interface similarity network derived from our PatchBag algorithm can recapitulate a high percent of the known interacting partners. Our results are comparable to the results of the algorithm presented by Cukuroglu *et al*.^49^, who applied the multiple protein structure alignment algorithm MultiProt^50^ to compare and cluster Protein-Protein Interfaces (PPIs). Furthermore, we demonstrate that PatchBag can identify homologous interfaces from randomly selected surface patches of the same size on the protein. Overall, PatchBag is highly efficient and can be used as an inverted index in future interface based search systems. We propose that PatchBag can help to identify similar interfaces regardless of their fold, providing a novel approach for protein function prediction based on their functional interfaces.

## Results

### A Vector Representation of Protein Interfaces for Efficient Comparison

**Figure 1:**
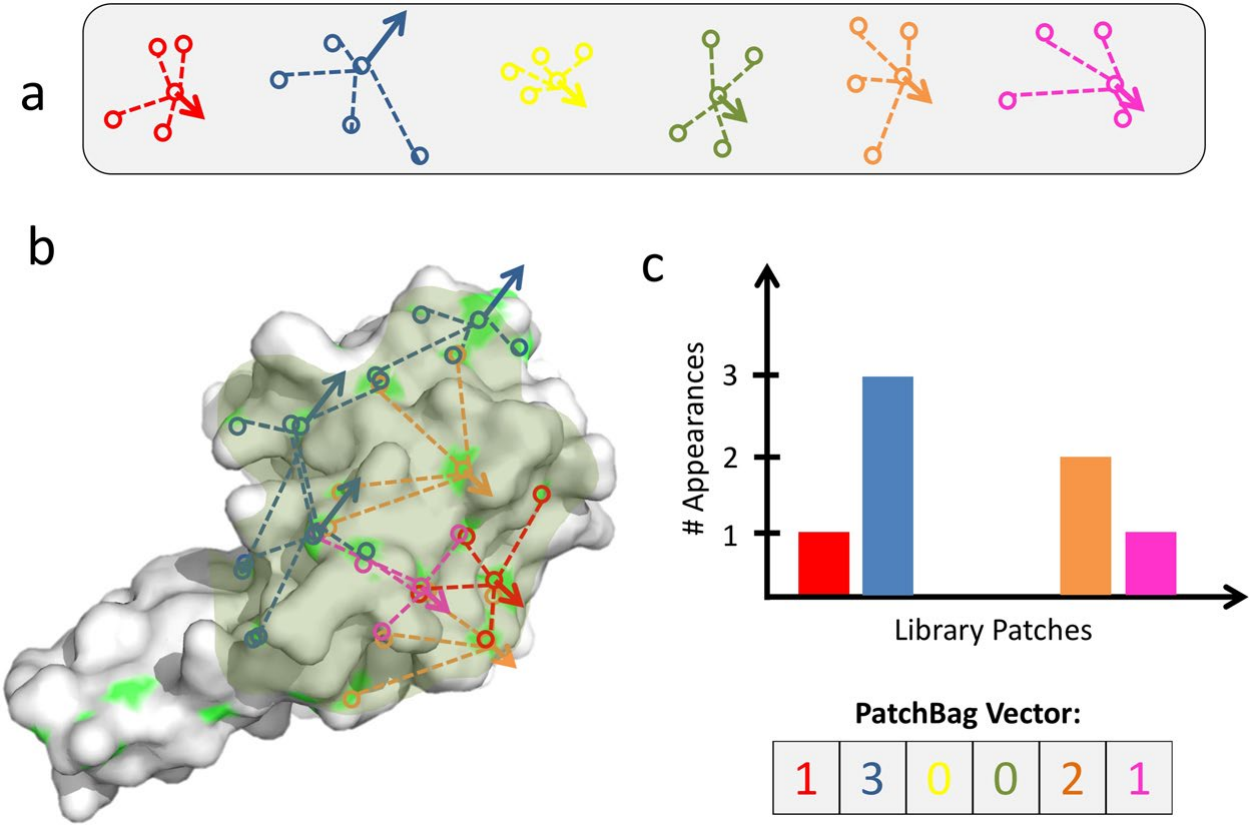
PatchBag overview: (**a**) Library of patches. All surface patches from a large training set of protein domains are extracted and clustered to form a library of patches. In this library, each surface patch has 5 C-Alpha atoms: a central one, and 4 additional C-Alphas that are the ones closest to it in space; a normal vector (represented by an arrow) anchored at the central C-Alpha atom, points to the outside of the patch. (**b**) Surface patches extraction. Given a protein structure, we describe all overlapping C-Alpha-centered patches in the complete surface, or only in parts of it, using the library patches that best approximate them. The protein surface C-Alpha atoms are marked in light green, and the interface area is shadowed in olive green. We illustrate only some non-overlapping surface patches, for a clearer view. (**c**) PatchBag vectors. We represent the protein surface by its PatchBag vector – a histogram, which counts for each library patch the number of times it best approximates a patch on the protein surface. We approximate the similarity between two protein surfaces by the cosine similarity of their corresponding PatchBag vectors.

PatchBag is a concise vector representation of protein surfaces or sub-surfaces (i.e., interfaces), based solely on the 3D structure of the protein. The protein surface is represented by all protein residues that are exposed to the solvent, while interfaces are defined as the subset of residue that interact with a specific partner (See Methods). An outline of the method is shown in Fig. 1. As a first step we build a representative library of surface patches on proteins. To this aim we define a surface patch of size *n* as a set of *n* 3D coordinates, representing the center of C-alpha atoms of an exposed residue calculated by the DSSP algorithm^51^ with a solvent accessibility threshold of 20% and its *n* − 1 nearest backbone residues, excluding the successor and predecessor residues along the backbone. We measure the distance between residues using Euclidean distance, and take *n* − 1 nearest neighbors with the minimum distance to the initial exposed residue. We eliminate successor and predecessor residues to capture the 3D space neighbors of the surface residue, rather than its backbone neighbors. In addition, we define the patch orientation by the direction of the normal vector pointing away from the protein core, calculated using the alpha shape algorithm^15^. We consider surface patches of sizes *n* = 4, 5, and 6 residues. For two surface patches of the same size, each represented by *n* C-alpha atoms, we define the distance between them as the minimal RMSD, calculated using Kabsch’s algorithm^52^ for all possible correspondences between the two sets of points. When comparing two surface patches, each represented by *n* C-alpha atoms, there are *n*! possible correspondences between the atoms in both patches (note that we do not enforce that the central C-alpha atoms of the two patches correspond to each other); we calculate the RMSD values of all possibilities in which the angle between the normal vectors is less than 90°, and take the minimum to be the true correspondence (for further details see Methods).

Next, we derive a library of patches by applying the surface patch similarity to a representative set of patches extracted from proteins surfaces. The patches library is essentially a set of patches resulting from clustering patches from a large set of representative protein patches (ranging from 4000 to 8000 randomly selected patches) from the SK.531 dataset (see Methods - Datasets). The clustering is based on the spatial distance between the patches, as measured by RMSD, and taking into account the surface orientation (distinguishing patches that their normal vector points to the protein center from patches that their normal vector points towards the protein surfaces). We first generate different patches libraries that vary in their number of elements (denoted library_size), starting from a randomly selected set of 5000 patches. We then cluster the patches to libraries of 20, 50, and 100 bags with different patch sizes (patch size ranging between 4 to 6 C-alpha atoms). We evaluate the quality of a clustering used to construct a library by the diameter of each cluster (favoring lower values) and the distance between clusters (favoring higher values) (see Methods for details). Table S1 lists the measures for the clustering results for the nine combinations of patch_size and library_size, generated from 5000 randomly selected patches. The characteristics of a representative library 〈6, 50〉 (patch_size = 6 and library_size = 50) are shown is Fig. S1. To test whether the results are dependent on the number of the patches selected to generate the libraries, we repeated the library generation procedure of the 6_50 library with 4,000, 6,000, 7,000 and 8,000 patches and recorded the inter- and intra- distances of each generated library. As demonstrated in Fig. S2, results are highly consistent when libraries were generated from 4,000 patches or from 8,000 patches. We thus chose to continue with the representative library 〈6, 50〉 generated from 5,000 randomly selected patches.

**Figure 2:**
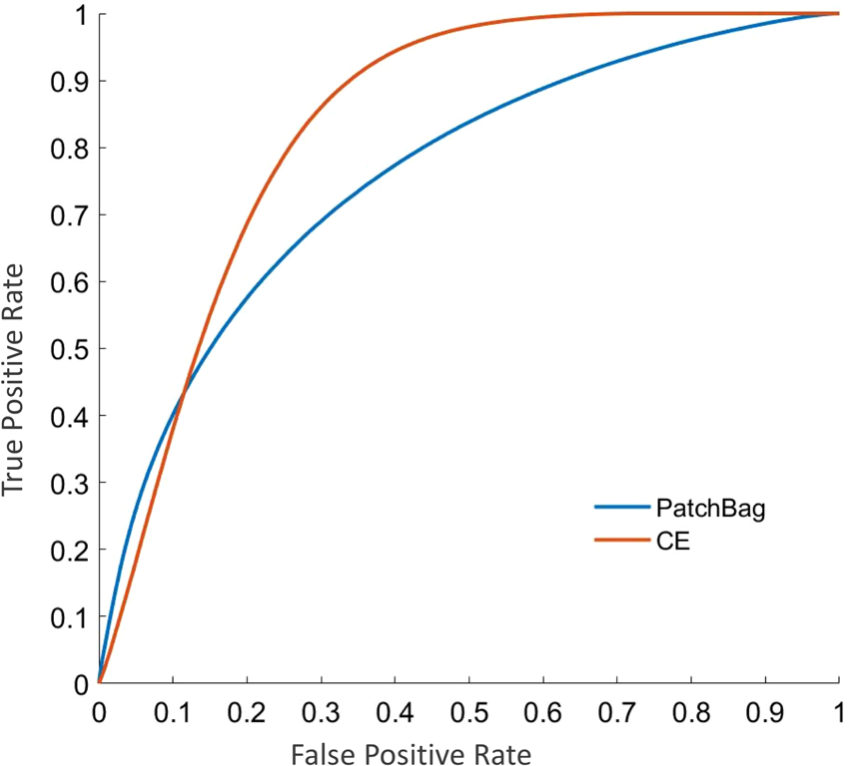
PatchBag performances on surface comparison. ROC plots demonstrating PatchBag results for all vs all domain surface comparisons and for all vs all structural comparisons using the CE structural alignment tool, both tools were tested on a dataset of 2,743 domains. As shown, PatchBag, that is based on the protein surface only, achieves an AUC of 0.76, which is comparable to the results attained with the well-trusted protein structural alignment method CE, that considers the entire domain fold (AUC of 0.84).

Further, to represent a protein surface/interface by a PatchBag vector, we consider the residues from the entire surface or the residues from a defined protein interface, for surfaces and interfaces, respectively, and extract all overlapping patches (as described in detail in the Methods section). Consequently, each patch extracted from the protein’s surface or interface is compared to the medioid of each bin in the library and is represented by the library patch most similar to it (See Fig. 1). Eventually, we generate a PatchBag vector, which describes the number of occurrences of these approximating library patches. If *k* is the library size, then the protein surface/interface will be represented by a vector of size *k* that counts for each library patch the number of times it is represented. We approximate the distance between two surfaces by the “PatchBag distance”, which is 1 – the cosine between their PatchBag vectors (see equation (2) in Methods). To measure the performance of PatchBag in evaluating protein surfaces similarity, we test it on a dataset of surfaces extracted from 2,743 30% sequence non-redundant protein domains from the KKL.2743 dataset (as described in the Methods section). Then, we evaluate the surface similarity by calculating all vs. all PatchBag scores. To assess the results we calculate the AUC (Area Under Curve) of the ROC (receiver operating characteristic) curve, where a pair of domains are considered similar if their Structure Alignment Score (SAS)^53^ is below a threshold T = 5 Å. We then compare PatchBag results to the AUC generated for structural comparison of the domains, using the protein structure alignment tool by incremental combinatorial extension (CE)^2^. Figure 2 presents the ROC plots of PatchBag (library size = 50, Patch_size = 6) for surface comparison vs. CE. for structural alignment tested on KKL.2743 protein domain dataset. As demonstrated, although PatchBag relies on information from the protein surface it can accurately detect similarities between domain structures, achieving an AUC of 0.76, compared to an AUC of 0.84 attained with a standard protein structural alignment method, such as the well-trusted method CE. Notably, the performance of PatchBag was not influenced by the library size or the patch size - see Fig. S3.

### A Network Approach to Validate Protein Interface Comparison

**Figure 3:**
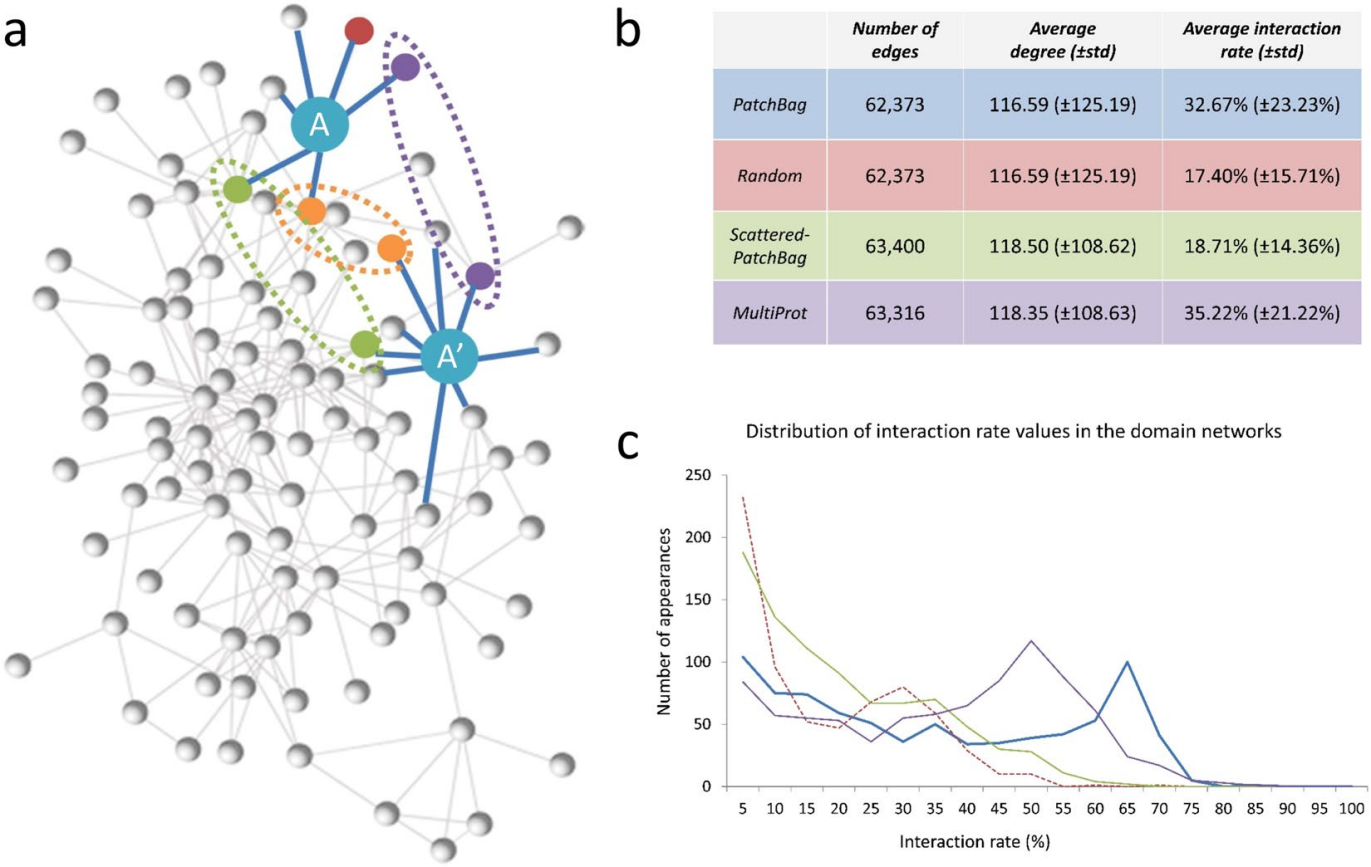
Interface Similarity Network (ISN). (**a**) The network’s nodes are 3DID domains: each interacting pair contributes two nodes, represented as two points (e.g., there are two blue nodes). Edges connect similar nodes, according to similarity criteria and a threshold value. Given a network, we count for each 3DID interacting pair 〈A, A′〉 the number of neighbors of A that interact with the neighbors of A′ (as documented in 3DID). The illustration shows the count for the blue 3DID pair: A′ has 9 similar interfaces (including the purple, yellow, and green ones), and A has 6 similar interfaces (including the purple, yellow, and green ones); hence there are 3 interacting neighbors (namely, the purple, yellow, and green). The interaction rate of domain A is 50% (3 out of 6 nodes are interacting), and the interaction rate of node A′ is 33.3% (3 out of 9 nodes are interacting). (**b**) Summary of PatchBag, Randon, Scattered-PatchBag and MultiProt ISNs properties: number of edges, average degree, average interaction rate and P-value of the difference in the interaction rate distribution compared to the PatchBag-ISN. (**c**) Interaction rate distributions of each ISN.

We use a network approach to assess the performance of interface comparison methods on a dataset of protein-protein interactions, 3DID, including 1070 interacting pairs^48^ (see Methods – 3DID.1070 datasets). For each protein among the protein-protein interacting pairs we define the interface residues based on the reported interacting residues, and extract their interface patches as described above and detailed in the Methods section. Our working hypothesis is that if two proteins have similar interfaces then their true partners are likely to have similar interfaces as well^54^. We define an Interface Similarity Network (ISN), where nodes represent protein interfaces and edges connect nodes representing similar interfaces. For a pair of interacting interfaces A and A′, we define the interaction size of the node A to be the number of neighbors of A in the ISN that interact with the neighbors of A′ in the ISN (Fig. 3a). The interaction rate of the node A is the ratio between its interaction size and its degree in the ISN. We analyze the overall distribution of interaction rates for all nodes in the ISN. As a control, we generate ten Random-ISNs with an identical degree distribution as the original network and compare the interaction rate distributions between the networks (see Methods for details)^55^.

In PatchBag-ISN, we measure the similarity between two interfaces by their PatchBag score. The number of nodes in the network is the number of domains – 1,070 (one interface per domain), the number of edges varies, depending on the threshold *T* for the PatchBag similarity score that we use; we consider *T* = 0.3, 0.4, 0.5. As expected, when using a higher threshold, the average interaction rate increases. Nonetheless, the distributions of the interaction rates are qualitatively similar for different PatchBag thresholds (Fig. S4a,b). For each threshold we compared the interaction rate distribution of the ISN to the corresponding Random-ISN using Mann-Whitney U test^56^. The most significant difference was found for *T* = 0.4 (p-value, 4.75 × 10^−48^), which we use in all the following tests.

As shown in Fig. 3b, the average interaction rate for the PatchBag-ISN is 32.67% (±23.23%). This is significantly higher than the average interaction rate for Random ISNs of the same number of nodes and edges 17.4% (±15.71%) (p-value of 2.17 × 10^−39^, Mann-Whitney U test). To verify that the difference between PatchBag-ISN and Random ISN does not depend of the random model chosen, we generate the random network in three different ways: (1) as a randomly rewired network generated by permuting the nodes. In other words, we shuffle the interacting pairs and rewire the structural neighbors while maintaining the degree distribution. (2) as a randomly rewired network without permuting the nodes; here, we maintain the interacting pairs while rewiring the structural neighbors. (3) as an Erdős network^57^, in which each edge is included with a probability p = 1/|E|). Figure S4c,d shows that the interaction rate in PatchBag-ISNs is significantly higher than in all other random networks.

To support that the PatchBag similarity is due to the similarity of the true interfaces, and not to the overall shape of the domains, we use a random set of surface residues as a control. We generate a Scattered-PatchBag-ISN, where similarity between interfaces is calculated based on the similarity between *N* randomly chosen scattered patches on the protein surface, where *N* is the number of residues on the original interface. The interaction rates depend on the node’s degree, which derives from the overall number of edges in the network. Thus, when comparing two networks generated by two comparison methods, it is important to set the threshold *T* such that the number of edges in each network is similar. In the Scattered-PatchBag-ISN we used *T* = 0.41, which yields 63,400 edges (comparable to 62,373 nodes in PatchBag-ISN with the threshold of T = 0.4). As shown in Fig. 3, the average degree is 118.50 (±108.62) and similar to the average degree of the PatchBag-ISN 116.59 (±125.19), however the average interaction rate is significantly lower: 18.71% (±14.36%) compared to 32.67 (±23.23) in the PatchBag-ISN (Fig. 3b, and full distributions in Fig. 3c, green and blue lines). The p-value of the difference between the interaction rates of the Scattered-PatchBag-ISN and the original PatchBag-ISN is 2.73 × 10^−32^ (Mann-Whitney U-test). Notably, the average interaction rate for the Scattered-PatchBag-ISN is higher than the equivalent value for the Random Networks, indicating that randomly chosen patches on the surface maintain some of the signal.

Finally, we compare the performance of PatchBag to that of the structural alignment method MultiProt^50^, customized to compare structural interfaces, as used by Cukuroglu *et al*.^49^, using the same network approach and set of residues as in the PatchBag-ISN. We construct the MultiProt-ISN from 63,316 edges (see Methods) with an average degree of 118.35 (±108.63) – similar to the average degree of the PatchBag-ISN. The average interaction rate of MultiProt-ISN is slightly higher than PatchBag-ISN, yet it has a similar distribution: 35.22% (±21.22%) (Fig. 3c, purple line). A summary of the attributes of each network and the interaction rate distributions are shown in Fig. 3b and c, respectively. Overall, these results indicate that our BOW approach, PatchBag, performs similarly to alignment-based approaches, such as MultiProt. Nevertheless, as described in detail below, PatchBag is orders of magnitude faster.

### Interface Homology Search

**Figure 4:**
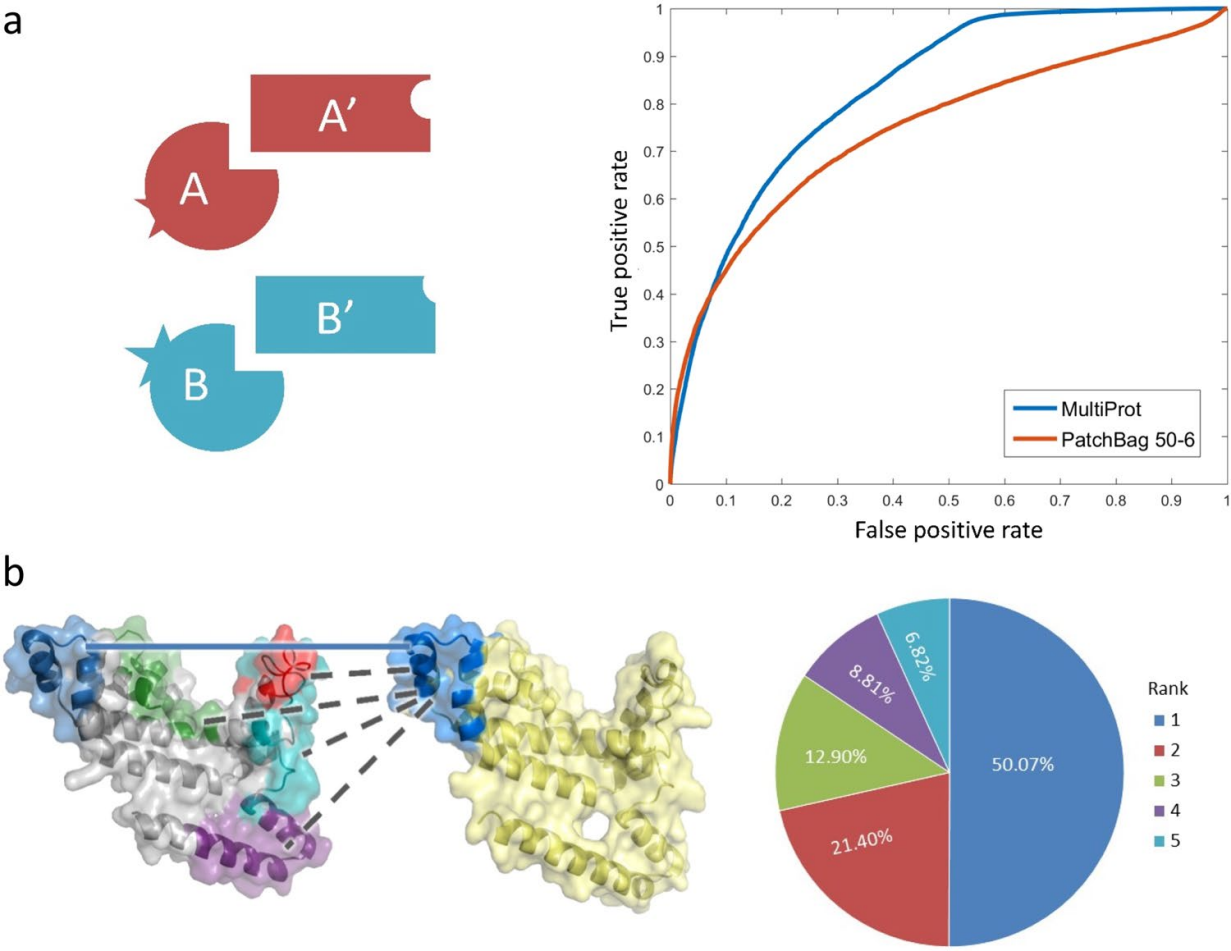
Interface recognition. (**a**) An illustration of the interface similarity proximity (left panel), where not only domains A and B are structurally similar, but their interacting domains A′ and B′ are similar as well. This approach filters cases where A and B have similar structures but different interfaces, as they bind different partners. As there is no standard measure to evaluate interface similarity, this similarity measure serves as our gold standard when comparing interfaces. For each protein interface Q we rank all other interfaces in the dataset in terms of their similarity to Q, and plot the ROC curve (right panel) of their PatchBag distance (red line) and MultiProt distance (blue line). PatchBag achieves an AUC of 0.75 and MultiPort achieves an AUC of 0.83, yet PatchBag is magnitudes faster. (**b**) An illustration of comparing a native interface to interfaces on a similar protein domain. The native interface is marked in blue and the rest are artificial interfaces, defined as a continuous sub-surface of the size of the native interface, with at least one residue which is not on the native interface. For each domain in the dataset, we generate 4 artificial interfaces. The pie chart presents the native interface rank results: we sort the interfaces by their PatchBag score and rank the native interface. Note that the rank can vary between 1 and 5. In 50% of the cases the native interface is ranked first, and in 71% it is ranked first or second.

Next, we assess how well PatchBag identifies reliably similar candidate interfaces within a database of interfaces. Let I_A_ and I_B_ be two interfaces on the surfaces of proteins A and B in the 3DID.1070 dataset. We consider I_A_ and I_B_ similar if two conditions hold: (1) A and B have the same PFam classification, and (2) A′ and B′, the interacting proteins of A and B, have the same Pfam classification as well (see Fig. 4a, left panel). We require both conditions, as there may be cases where one side of the interface - proteins A and B, are similar, while the other - proteins A′ and B′, are not, suggesting the interfaces are different (as illustrated in Fig. S5). To test PatchBag, for each protein interface Q we rank all other interfaces in the dataset in terms of their similarity to Q, using the PatchBag distance between Q and all other interfaces in the dataset. As shown in Fig. 4a (right panel), PatchBag (red line) achieves an AUC of 0.75 in this similarity test. When conducting the same test using MultiPort, it achieves an AUC of 0.83 (blue line). Notably, while multiport performs better than PatchBag on this set it is important to note that the running times are dramatically different: on this dataset running MultiProt took 5 hours, compared to less than a second for running PatchBag (see details below).

A compelling feature of PatchBag is that it can describe sub-surfaces of a protein, and can thus be used to distinguish between a part of the surface that is the functional interface from other parts of the protein surface. To test if PatchBag can recognize the native interface among a collection of interfaces on the same protein structure, we generated a set of four decoy interfaces per each protein domain. A decoy interface is a continuous sub-surface of the same size as the native interface, and differs in at least one residue from the native interface. As illustrated in Fig. 4b (left panel), we compare the PatchBag vectors of the native and decoy interfaces of domain A to the PatchBag vector of the native interface of domain B, and rank them by their PatchBag score, expecting the native interface to be ranked first. As shown in Fig. 4b, in 51% of the cases the native interface is ranked first (compared to 20% expected by chance) and in 71% of the cases, the native interface is ranked among the top two (compared to 40% expected by chance).

### Running Times

PatchBag merely compares two vectors; hence, after the database is preprocessed, pairwise surface or interface comparison is very fast. We ran our experiments on the 3DID.1070 dataset (see Methods – Datasets) using a computer running 8 Intel i7-2600K 3.40 GHz cores to run PatchBag in this dataset. The preprocessing of a single structure includes extracting its surface or interface and calculating its PatchBag vector according to the selected library. The preprocessing step takes 9 seconds per surface/interface on average, depending on the number of residues in the overall structure and on the surface or the interface. The preprocessing step of a dataset of 1,070 protein interfaces took 2.7 hours, once we had the PatchBag vectors, comparing all-vs.-all (571,915 pairs) took around one second. Running MultiProt for all-vs.-all comparison on the same computer took 5 hours, or 0.03 seconds per single comparison. Using MultiProt, as there is no pre-processing option, the querying time of the 3DID.1070 dataset is 32 seconds, and it will grow linearly with the size of the dataset.

## Discussion

PatchBag is a novel geometry-based method to compare protein surfaces and interfaces, based on the bag-of-words (BOW) approach. BOW is widely used in applications of automated search: For example, in textual search for efficiently comparing documents^45^ and in computer vision for comparing images^58^. The BOW approach has also been employed to compare protein sequences, where a word is a sequence *k*-mer^46^, or to protein structures, where a word is a short structural fragment on the protein backbone^47^. In PatchBag, the words are small structural patches on the protein surface, defined by the coordinates of 4–6 C-alpha atoms that are structural neighbors of a central exposed residue. The pitfall in defining a protein surface using a surface accessibility threshold is that proteins tend to have structural fluctuations. Sometimes, even a minor fluctuation can cause a buried residue to become exposed, and vice versa. Such an event is very common, and alters the surface structure. Defining a surface patch by a central exposed residue along with its spatial neighbors, which are not necessarily solvent accessible, renders PatchBag less sensitive to small structural fluctuations and thus more robust.

PatchBag infers interface similarity by the relative frequency of different structural patches existing on the interface. Another valuable characteristic of BOWs is that they can be used within an inverted index data structure, which stores each interface according to its patches, rendering search even faster. Interestingly, even though PatchBag, as other BOW-based methods, completely ignores the order of the words (patches), it can still recognize interface similarities. PatchBag, unlike other interface comparison methods^23,28,43^, is sequence independent, as it only considers the three dimensional coordinates of the C-alpha atoms of the interface residues. This allows us to find interface similarities among non-homologous proteins. Moreover, as opposed to some state-of-the-art interface comparison methods, such as the method proposed by Brender and Zhang^59^, PatchBag does not require a prior structural alignment. This is especially useful to identify similar interfaces even if the overall structure of the proteins is different, thus enabling us to identify structurally similar interfaces lying on completely different protein folds. The strength of PatchBag lies in its robustness to represent any type of protein interface (protein-, nucleic acid- or ligand- binding interfaces), and infer similarity of any kind. Most existing interface comparison methods are designed to compare the paired-interface of protein-protein complexes, which includes the interface of two interacting partners^29,54^. Paired-interface search is useful for docking tasks, or template based PPI search^60^, however, it is less suitable for finding similarities between protein interfaces that bind other ligands, such as nucleic acids. Other methods are uniquely designed to find similarities among small ligands binding interfaces or pockets^13,39,61^. The latter methods identify similarities between binding sites in different proteins that bind the same ligands, yet they are not applicable for searching protein interface similarity in general, as they require the convexity of the interface, which is not necessarily the case in PPIs.

The uniqueness of PatchBag is that it allows comparing any given region on a protein surface to any other surface region without any requirements or assumptions on the interfaces of interest, such as being on homologous proteins or knowing the interacting partner of the interfaces. The lack of compatible computational methods to compare interfaces led us to find a unique approach to evaluate PatchBag’s performance. Our approach assumes that two similar interfaces tend to have similar partners. Thus, we build an Interface Similarity Network (ISN), in which the nodes are protein interfaces, and edges connect similar interfaces according to a similarity measure. Protein similarity networks are commonly used to explore protein sequence space^62^ or structure space^63,64^. We take advantage of protein similarity networks to validate our new interface similarity measure. Specifically, we evaluate PatchBag  by its ability to recapitulate known interactions from PPI data. The ISN approach allows us to examine a large dataset of interfaces, instead of studying a few examples. We further use this approach to compare PatchBag to MultiProt^50^, which has been used to compare paired-interfaces^49^, namely, the contact region between two interacting proteins. Here, we employ MultipProt to single interfaces and show that PatchBag performs on par with it though significantly more efficiently. We thus propose that ISNs can also serve to evaluate other interface similarity methods.

A great advantage of PatchBag is its pre-processing step, which allows us to calculate the PatchBag vectors of a large dataset in advance. Then, querying a pre-processed dataset using a new protein interface will take only a few seconds to prepare the PatchBag vector of the query interface, and a few nanoseconds to compare it to the entire dataset, as calculating the “PatchBag distance” of vectors is a rapid computation. Thus, whether the pre-processed dataset is of size 1,000 interfaces or 100,000 interfaces, its querying time remains the same, and it depends mainly on the queried interface, and less on the dataset size. PatchBag can be further employed to index interfaces from the PDB, as well as intra-domain interfaces, that is, areas within a domain that are often found to be evolutionary related to domain-domain interactions^65^. This index can be used as a powerful interface search engine that given a query interface will instantly filter all potentially similar interfaces. On the filtered set, which only contains a fraction of the interfaces in the database, one could apply a more accurate (yet computationally expensive) method to refine the results. Such a tool would be useful for template based PPI prediction, by the process defined in the work of Muratcioglu *et al*.^66^. It can also be applied to search for a specific interface among a set of candidate interfaces on a protein’s surface, for example – for searching a DNA and RNA binding interfaces, given a set of candidate binding interfaces on a protein surface, or for searching a ligand binding interface from a list of potential interfaces given by a pocket prediction tool, such as CASTp^35^. By allowing a fast, sequence- and fold- independent search in large datasets of functional surfaces, PatchBag can boost the current comprehension of protein function, and reveal novel evolutionary relationships between protein surfaces and interfaces, which are yet unidentified due to lack of sufficient and efficient computational tools for surface comparisons.

## Methods

### Datasets

#### SK.531 - Patches libraries

We use a dataset which consists of 609 protein domains^44^. In this dataset each domain was taken from a different SCOP^67^ family, from 72 SCOP superfamilies and the structure similarity of each pair is less than a Z-score of 3.8 by the Combinatorial Extension (CE)^2^. To prepare the patches libraries we filter structures with less than 50 residues. In addition, we exclude domains that were removed from SCOP version 1.75B, structures solved by NMR, and structures with crystallographic resolution of 3.0 Å or worse. This results in 531 sequence non-redundant protein domains.

#### 3DID.1070 Protein-Protein interfaces dataset

The 3DID dataset is a catalogue that contains all domain-domain interactions from the PDB^48^. We filter out domains with 80% sequence identity or more using the CD-HIT software^68^ and take only sequences longer than 50 residues. Then, we extract the interfaces from each domain within the domain pairs by considering only the interacting residues, as defined in the 3DID database. Notably, we consider only pairs where both interfaces are larger than 15 residues. Overall, we have 535 such pairs, that is, 1070 interfaces.

#### KKL.2743 Structural similarity dataset

For surface comparison we use a dataset of 2,743 structurally aligned protein domains from CATH generated in a previous study^69^, where the SAS (Structural Alignment Score) of all vs. all alignments were recorded. SAS score is the ratio of the RMSD and alignment length times 100^53^. In our gold standard, two domains are considered similar if their SAS score is below a threshold T = 5 Å, a threshold on which sequence alignment similarity yields an AUC of 0.5^47^.

#### Protein surfaces, interfaces and surface patches definitions

The protein *surface* is the collection of surface accessible residues. We calculate the exposed residues using the DSSP algorithm^51^ with surface accessibility threshold of 20%. The protein *interface* is a subset of the protein surface, which includes the residues that interact with a substrate. In the 3DID dataset the interface residues are provided in the data files. We define a surface *patch* by a central exposed C-alpha atom and its nearest C-alpha neighbors, excluding the C-alpha atoms of the successor and predecessor residues along the polypeptide chain. In addition, we define the patch orientation by the direction of the normal vector pointing away from the protein core. To calculate the normal vectors we compute the alpha shape triangulation^15^ of the protein surface, and use the triangulation facets to compute the normal of each residue.

### Generating the patch libraries

For each given protein domain we extract the surface residues using DSSP^51^, and consider its surface patches. Then, we approximate each patch by its most similar counterpart in a library of patches. We randomly selected 4000, 5000, 6000, 7000 and 8000 patches from the 60,070 patches on the proteins in the SK.531 dataset (see Methods – Datasets), and used the *k*-means++ algorithm^70^, in each case clustering them into *k* = 20, 50, and 100 clusters. The mediods (the surface patches closest to the centroids according to the surface patches similarity score) of each cluster were collected to form the surface patch library.

Given that the *k*-means++ algorithm initializes the mediods randomly it is expected that any two runs of the algorithm on the same data would yield different results. To choose the best clustering result we used the Dunn clustering score^71^, which is the ratio between the minimal inter-cluster RMSD to maximal intra-cluster RMSD, shown in equation (1).

1 $$Clustering\,Score=\frac{mi{n}_{1\le i\le k}\{mi{n}_{1\le j\le k,i\ne j}\{d(i,j)\}\}}{ma{x}_{1\le m\le k}\{D(m)\}}$$

Equation (1): *d*(*i*, *j*) is the inter-cluster distance between cluster *i* and cluster *j*, measured by the distance between their mediods, and *D*(*m*) is the intra-cluster distance of cluster *m*, measured by the mean distance between each cluster member and its mediod. We clustered the data 50 times and chose the medoids with the maximal Dunn score as our patches library.

### Calculating protein surfaces or interface similarities using PatchBag

Given a library *L* < *patch_size*, *library_size* > , we characterize a protein surface or subsurface *P* by its PatchBag vector of *library_size* entries; the vector represents the number of times each library-patch best approximates a surface patch in *P*. We calculate the similarity between two protein surfaces by the cosine distance of their corresponding PatchBag vectors – see equation (2). We refer to 1 - the cosine angle between two PatchBag vectors as “PatchBag distance”.

2 $$Patch\,Bag\_Distance\,({P}_{1},{P}_{2})=\,1-\frac{{P}_{1}\cdot {P}_{2}^{T}}{\sqrt{{P}_{1}\cdot {P}_{1}^{T}}\cdot \sqrt{{P}_{2}\cdot {P}_{2}^{T}}}$$

Equation (2): *P*_1_ and *P*_2_ are PatchBag vectors of size *library_size*, describing two protein surfaces.

### Comparing Interface Similarity Networks (ISNs)

Interface Similarity Network, is a network in which nodes represent protein interfaces and edges connect nodes representing similar interfaces. In PatchBag-ISN each pair of interfaces (nodes) were connected by an edge if their “PatchBag distance” was below threshold *T (T* = 0.4). Overall, the PatchBag-ISN yielded 62,373 edges. Note, that when comparing ISNs generated by two different comparison methods, it is important to set the threshold *T* such that the number of edges is similar. Hence, to keep the number of edges similar in all ISNs, in Scattered-PatchBag-ISN, T was set to 0.41 yielding 63,400 edges, and in MultiProt-ISN we used *T* = 6.1, resulting in 63,316 edges.

Given that domains 〈A, A′〉 are interacting according to the 3DID database, we measured the interaction size of node A, that is, the number of neighbors of A whose interacting partner is in the neighbors group of A′. Finally, we recorded the interaction rate, which is the proportion between the node’s interaction size and its degree.

A stand-alone version of PatchBag software is avaialble via request.

## Electronic supplementary material

Supplementary Information
