# Supplementary material for: A Novel Geometry-Based Approach to Infer Protein Interface Similarity
Source: Sci Rep. 2018 May 29;8:8192. doi: 10.1038/s41598-018-26497-z (PMC5974305; doi:10.1038/s41598-018-26497-z)
Supplement: Supplementary file 1 — Supplementary Information [file 41598_2018_26497_MOESM1_ESM.pdf]

A Novel Geometry-Based Approach to Infer Protein Interface Similarity

Inbal Budowski-Tal, Rachel Kolodny and Yael Mandel-Gutfreund

Properties of Clustering Results for Different Sizes of Library and Patch

Patch Size	Library Size	Dunn Score	Mean Inter-Cluster RMSD (±std) (Å)	Mean Intra-Cluster RMSD (±std) (Å)	Significance* of the Difference between the Inter and Intra RMSD
4	20	0.61	0.11 (±0.024)	0.22 (±0.068)	1.89×10^{-11}
	50	0.37	0.10 (±0.016)	0.22 (±0.059)	4.38×10^{-31}
	100	0.31	0.08 (±0.024)	0.24 (±0.082)	6.55×10^{-62}
5	20	0.59	0.16 (±0.024)	0.24 (±0.051)	1.47×10^{-10}
	50	0.46	0.14 (±0.030)	0.28 (±0.074)	2.69×10^{-29}
	100	0.30	0.13 (±0.024)	0.26 (±0.053)	7.88×10^{-61}
6	20	0.59	0.20 (±0.030)	0.29 (±0.045)	1.33×10^{-10}
	50	0.42	0.19 (±0.022)	0.30(±0.069)	1.04×10^{-28}
	100	0.33	0.17 (±0.034)	0.31 (±0.071)	1.41×10^{-61}

*calculated by the p-value of Mann Whitney U test

Table S1: Properties of clustering results for different sizes of library and patch.

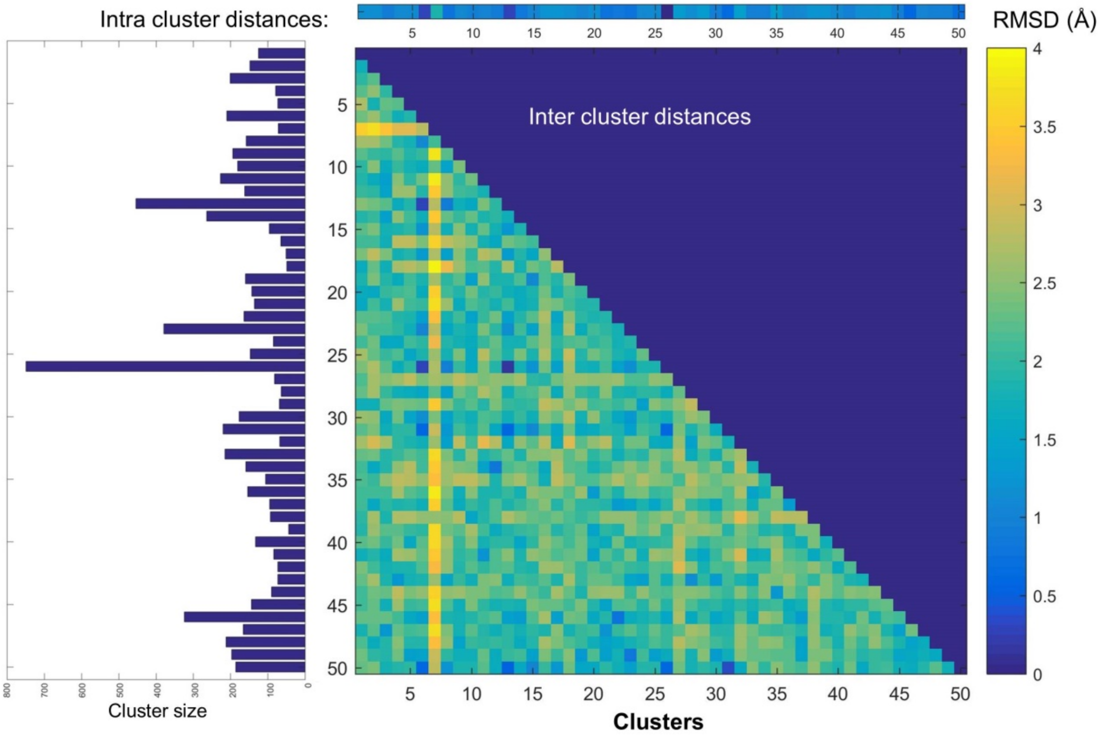

<u>Figure S1: Characteristics of 6_50 library</u>. The library was created by clustering 5,000 surface patches of size 6 into 50 clusters. The half matrix heat map represents the all vs. all RMSD between the cluster medioids (inter-distance) – colorbar is on the right. At the bottom there is a vector heat map of the mean RMSD between each cluster member and the medioid (intr-distance), applied on the same colorbar. Note that the intra-distance is much lower than the inter-distance. On the left there is a bar plot of the cluster size.

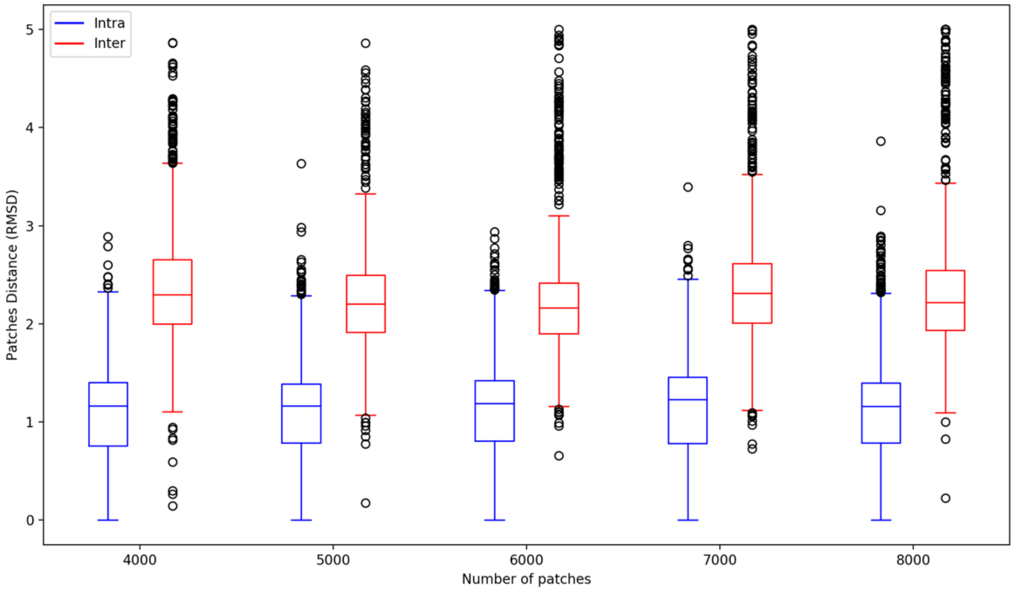

<u>Figure S2: Intra- and inter- cluster distances distribution of the 6_50 library conducted from different sets of randomly selected patches.</u> The patches library is built from 4,000, 5,000, 6,000, 7,000 and 8,000 surface patches of size 6, clustered into 50 clusters. The distribution of the intra-distances, that is, the distance between each patch and its medioid is shown in blue. The distribution of the inter-distances, that is, the distance between all the clusters medioids is shown in red.

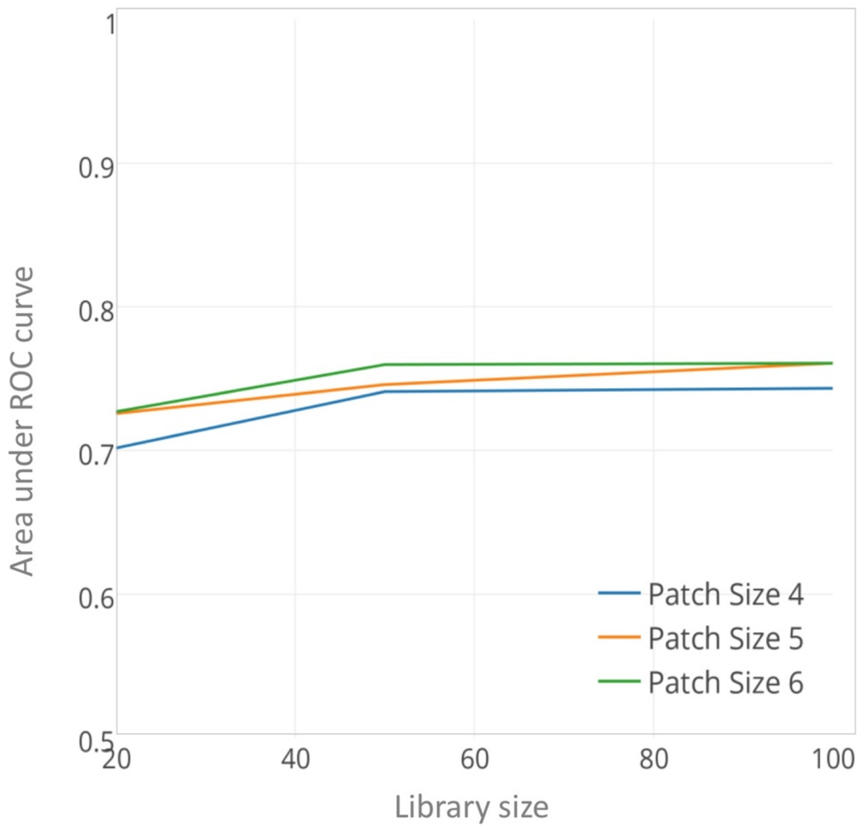

<u>Figure S3: PatchBag's performance with different parameters.</u> We used area under ROC curve on a gold standard of 2,743 structurally aligned protein domains to measure PatchBag's performance. The plot shows the performance of PatchBag on the protein surface using a library of library_size=20, 50, 100 patches along the x-axis, and patch_size=4, 5, 6 amino acids in blue, red and green accordingly. The y-axis refers to the AUC under ROC curve of PatchBag obtained using each library.

a

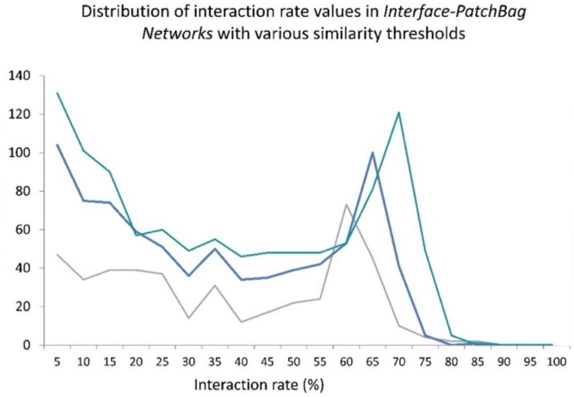

b

PatchBag similarity threshold	Number of edges	Average degree (±std)	Average interaction rate (±std)
0.3	29,568	55.27 (±85.98)	39.39% (±28.11%)
0.4	62,373	116.59 (±125.19)	32.67% (±23.23%)
0.5	120,601	225.42 (±157.28)	35.74% (±24.28%)

c

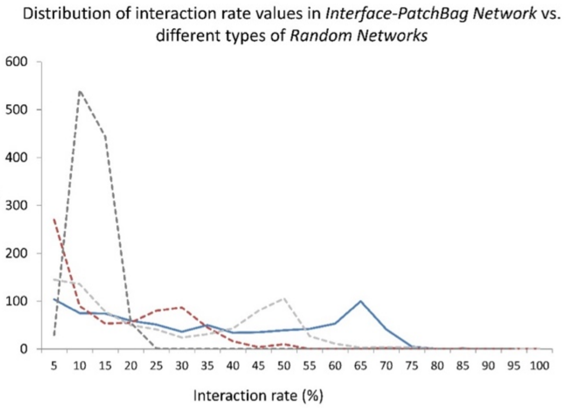

d

	Number of edges	Average degree (±std)	Average interaction rate (±std)
PatchBag-ISN	62,373	116.59 (±125.19)	32.67% (±23.23%)
Same Degree Distribution with Pairs Shuffle	62,373	116.59 (±125.19)	17.40% (±15.71%)
Same Degree Distribution without Pairs Shuffle	62,373	116.59 (±125.19)	24.55% (±19.22%)
Uniform Degree Distribution	62,373	118.34 (±10.08)	11.00% (±2.90%)

Figure S4: PatchBag true vs. random network analysis. **(a)** Distribution of interaction rates for PatchBag-ISNs with similarity thresholds 0.3, 0.4 and 0.5. As expected, higher thresholds yield networks with more edges, thus the number of appearances increases. Note that as the number of edges increases, the chance of having a higher interaction rate increases, thus there is a slight shift to the right as the threshold increases. **(b)** A summary of the PatchBag-ISNs properties, including number of edges, average degree and interaction rate, and the P-value of the distribution difference from the corresponding random ISN. The best P-value was retrieved with similarity threshold 0.4 **(c)** Distribution of interaction rates for PatchBag-ISN and 3 random ISNs. We defined random networks according to three models: 1- rewire the PatchBag-ISN edges while preserving the overall degree distribution and shuffle the node identifiers. Here, a node in the random network will not necessarily have the same degree as in the original network. 2- rewire the true network's edges while preserving the overall degree distribution. 3- random network with the same number of edges as in the original network. Using this model, edges are distributed equally so the degree of each node equals |E|/|V|. As expected, the interaction rate distribution of the random networks of each model are different and lower than the PatchBag-ISN. **(d)** Summary of the Random-ISNs properties. Note that the P-values of the all random models indicate that the interaction rates are significantly lower than the PatchBag-ISN.

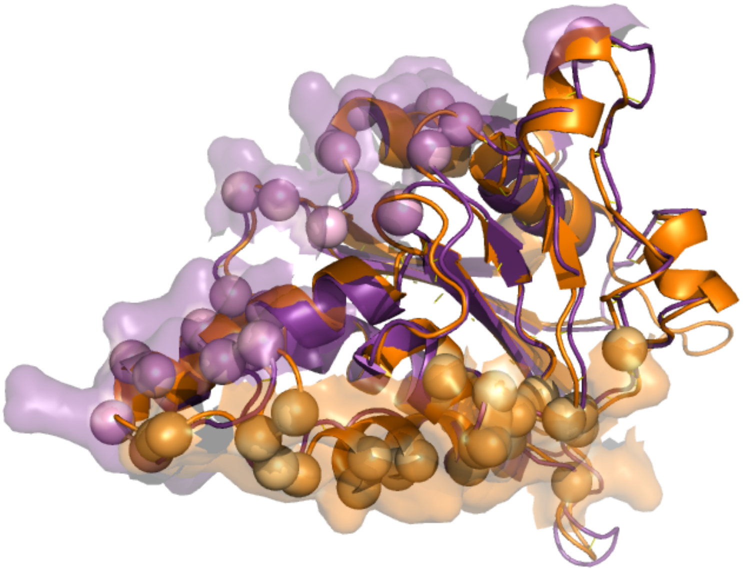

<u>Figure S5: exceptions in the evaluation scheme.</u> Two superimposed domains from the 3DID dataset, with an identical Pfam classification: ATP synthase alpha/beta subunits, and yet their interfaces are different. PatchBag recognizes the difference between the interfaces with a high cosine distance of 0.64.
